# Mercury in Vulcanized Rubber Plates

**Published:** 1870-06

**Authors:** Jas. B. Hodgkin


### ARTICLE II.

### *Mercury in Vulcanized Rubber Plates.*

By Jas. B. Hodgkin, D. D. S.

As an item concerning the "mercury contained in dental plates made of India Rubber," has attracted some attention, I beg leave to offer a few statements with regard to the same, to relieve the minds of the very large class of persons who having been unfortunate enough to lose the organs provided for them by nature, and who being dependent on the dentist for substitutes, have been furnished with teeth mounted on plates made of India Rubber. I say "the very large class," for it is doubtless true that nine tenths of those wearing artificial substitutes for the natural teeth have rubber plates.

The mercury so-called, is not mercury in fact, any more than cider is vinegar, or starch is sugar, for while it is a compound of mercury, yet any chemist knows that the poisonous qualities of many simple bodies are altered or rendered inert by chemical manipulation. "Vermilion" is the preparation necessarily used by the manufacturers of India Rubber to give that article the ordinary red tint. This is the "sulphuret of mercury," "Hg. S," containing equivalent proportions of mercury and sulphur, an insoluble compound under ordinary circumstances. The quantity used in an ordinary set of teeth is from 30 to 60 grains, about one fourth or one fifth of the bulk of the plate. This is combined with the rubber, while the latter is in a plastic condition, and the compound hardened by the dentist as it is worked up by him. When thus hardened the resulting product known as "Vulcanite" is a firm, tough substance, familiar to the public in another form and color, as used in handles for knives, combs and the thousand uses to which this very extraordinary and most useful article is devoted.

We have then in a rubber dental plate, about on an average 45 grains of sulphuret of mercury firmly combined with rubber, in such a form that it cannot be reduced to the metallic or soluble condition, except by a degree of heat sufficient to sublime the mercury, (which takes place at 662 degress F., and this of course could not occur in the mouth, when it is remembered that water boiles at 212.) No other change can occur but this, and this leaves but one other source of danger of mercurial poisoning, and that would be from the wearing away of the plate and the consequent swallowing of the particles thus worn off. As these plates last for from ten to fifteen years and then do not wear out, but fail from some molecular change in the rubber, it will be apparent that this danger is not great. I have repeatedly examined rubber plates that have been worn for a year, and found the original polish not worn off.

I trust that what I have thus hastily written will put at ease some who have fancied themselves in danger of being poisoned—a danger altogether in the imagination, or invented by interested parties. The fact that countless thousands of persons of various ages and sexes have been wearing such plates for years with comfort; is a suffcient answer to this question of poisoning.

It may not be a miss by the way to add that a great many good people really do not know that they are taking mercury when they are, and blue pills, calomel and the like are swallowed under high sounding names very often; while the good lady who prescribes Ayer's pills or 888's thinks these are harmless vegetable compounds, it is perhaps as well, for physicians know that mercury is yet as it was named long years ago, "the strong man among remedies."

